# Expression and testing in plants of ArcLight, a genetically–encoded voltage indicator used in neuroscience research

**DOI:** 10.1186/s12870-015-0633-z

**Published:** 2015-10-12

**Authors:** Antonius J.M. Matzke, Marjori Matzke

**Affiliations:** Institute of Plant and Microbial Biology, Academia Sinica, 128, Section 2, Academia Road, Nangang District, Taipei 115 Taiwan

**Keywords:** ArcLight, Electrical signalling, Genetically-encoded voltage indicator, pH-sensitive indicator, Super ecliptic pHluorin

## Abstract

**Background:**

It is increasingly appreciated that electrical controls acting at the cellular and supra-cellular levels influence development and initiate rapid responses to environmental cues. An emerging method for non-invasive optical imaging of electrical activity at cell membranes uses genetically-encoded voltage indicators (GEVIs). Developed by neuroscientists to chart neuronal circuits in animals, GEVIs comprise a fluorescent protein that is fused to a voltage-sensing domain. One well-known GEVI, ArcLight, undergoes strong shifts in fluorescence intensity in response to voltage changes in mammalian cells. ArcLight consists of super-ecliptic (SE) pHluorin (**pH**-sensitive f**luor**escent prote**in**) with an A227D substitution, which confers voltage sensitivity in neurons, fused to the **v**oltage-**s**ensing **d**omain of the voltage-sensing phosphatase of ***C****iona****i****ntestinalis* (Ci-VSD). In an ongoing effort to adapt tools of optical electrophysiology for plants, we describe here the expression and testing of ArcLight and various derivatives in different membranes of root cells in *Arabidopsis thaliana*.

**Results:**

Transgenic constructs were designed to express ArcLight and various derivatives targeted to the plasma membrane and nuclear membranes of *Arabidopsis* root cells. In transgenic seedlings, changes in fluorescence intensity of these reporter proteins following extracellular ATP (eATP) application were monitored using a fluorescence microscope equipped with a high speed camera. Coordinate reductions in fluorescence intensity of ArcLight and Ci-VSD-containing derivatives were observed at both the plasma membrane and nuclear membranes following eATP treatments. However, similar responses were observed for derivatives lacking the Ci-VSD. The dispensability of the Ci-VSD suggests that in plants, where H^+^ ions contribute substantially to electrical activities, the voltage-sensing ability of ArcLight is subordinate to the pH sensitivity of its SEpHluorin base. The transient reduction of ArcLight fluorescence triggered by eATP most likely reflects changes in pH and not membrane voltage.

**Conclusions:**

The pH sensitivity of ArcLight precludes its use as a direct sensor of membrane voltage in plants. Nevertheless, ArcLight and derivatives situated in the plasma membrane and nuclear membranes may offer robust, fluorescence intensity-based pH indicators for monitoring concurrent changes in pH at these discrete membrane systems. Such tools will assist analyses of pH as a signal and/or messenger at the cell surface and the nuclear periphery in living plants.

**Electronic supplementary material:**

The online version of this article (doi:10.1186/s12870-015-0633-z) contains supplementary material, which is available to authorized users.

## Background

Growth, development and appropriate responses to the environment require electrical controls and networks acting at multiple levels of organization within cells, tissues and whole organisms [1–3]. At the cellular level, changes in transmembrane potentials (electrical voltage gradients) and ion fluxes comprise an extensive system of bioelectrical communication that is integrated with molecular, chemical and mechanical signalling pathways [2, 4]. Together with classical methods for monitoring membrane potentials such as microelectrodes and patch clamp, a new generation of electrophysiological tools is being developed based on the concept of light-based or optical electrophysiology [4, 5]. An important group of these new tools consists of genetically-encoded, protein-based voltage indicators [6–8].

Genetically-encoded voltage indicators (GEVIs) are composed of a fusion between a fluorescent protein (reporter) and a voltage-sensing domain (detector) [8]. GEVIs have been developed by neurobiologists over the last two decades as a non-invasive method to optically monitor changes in transmembrane potential in single and multiple neurons and other cell types [6–9]. One type of GEVI is based on Förster resonance energy transfer (FRET) between a pair of fluorescent proteins joined to a membrane-spanning voltage-sensing domain. Changes in membrane potential are thought to act through the voltage-sensing domain to induce more favourable alignment of the two fluorescent proteins, resulting in increased FRET efficiency [8–10]. By contrast, in monochromatic GEVIs, a transmembrane voltage-sensing domain is fused to a single fluorescent protein that reacts to a voltage change by showing alterations in fluorescence intensity. This has been proposed to result when membrane depolarization triggers movement of the voltage-sensing domain, resulting in deformation of the linked fluorescent protein in a manner that reduces fluorescence intensity [8].

One intensity-based GEVI is ArcLight [11, 12], which consists of super-ecliptic (SE) pHluorin (**pH**-sensitive f**luor**escent prote**in**) [13, 14] containing an A227D substitution conferring voltage sensitivity in neurons [11] and the voltage-sensing domain of the voltage-sensing phosphatase of *Ciona intestinalis* (Ci-VSD) [15]. The fluorescence intensity of ArcLight has been reported to change significantly in response to voltage changes at the plasma membrane in mammalian cells [12]. In one study using human embryonic kidney (HEK293) cells, the fluorescence intensity of ArcLight decreased 35 % in response to a membrane depolarization of 100 mV [11].

One advantage of GEVIs as voltage indicators is that they can be fused to defined membrane targeting motifs, thus allowing electrophysiological analysis of internal cellular membranes that are largely inaccessible to classical tools for measuring membrane potential. Although the membrane potentials of multiple cells can in principle be measured using microelectrode arrays [16, 17], GEVIs also permit noninvasive detection of simultaneous changes in membrane potentials in populations of cells in intact tissues and organs [6].

We are interested in using GEVIs to study coordinated changes in the electrical potentials of plasma membranes and nuclear membranes of plant cells in response to environmental and developmental stimuli. Owing to their low background fluorescence and interesting developmental features, root cells provide a good experimental system for evaluating the feasibility of GEVIs to study the electrical behavior of different membrane systems in living plants [18]. We described previously the generation of transgenic *Arabidopsis thaliana* (*Arabidopsis*) plants expressing FRET-based GEVIs in root cells [19]. FRET-based GEVIs are stably expressed and well-tolerated by *Arabidopsis* and a recent study documents the successful use of Mermaid FRET sensors to monitor membrane voltage changes in response to exogenous application of potassium in a plant system [20]. In view of former findings for ArcLight in mammalian cells showing large shifts in fluorescence intensity in response to voltage changes [11, 12], we have assembled and introduced into *Arabidopsis* constructs encoding ArcLight and several derivatives targeted to the plasma membrane and nuclear membranes of root cells. Here we describe the results of experiments designed to assess changes in the fluorescence intensity of ArcLight and derivatives situated in these two membrane systems in response to external ATP (eATP) and other stimuli expected to trigger changes in transmembrane potential [21].

## Results

### Transgenic Arabidopsis plants expressing GEVIs and derivatives in root cells

**Fig. 1:**
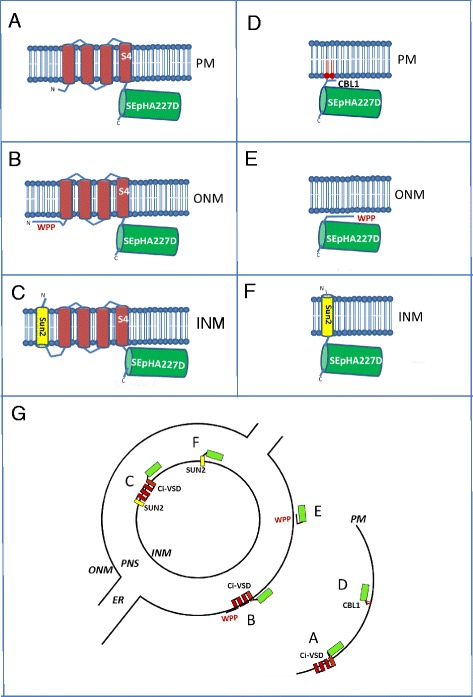
Diagrams of GEVIs and derivatives used in this study and predicted membrane localizations. GEVIs include: **A** ArcLight, which consists of SEpHluorinA227D fused to the Ci-VSD (transmembrane domains indicated as red bars with the voltage-sensing domain in S4); **B** ArcLight fused at the N-terminus to outer nuclear membrane (ONM)-tethering sequence WPP; **C** ArcLight fused at the N-terminus to inner nuclear membrane (INM) transmembrane protein SUN2. The derivatives, which do not contain Ci-VSD, include: **D** SEpHluorinA227D fused to the plasma membrane (PM)-tethering sequence CBL1; **E** SEpHluorinA227D fused at the N-terminus to WPP; **F** SEpHluorinA227D fused at the N-terminus to SUN2. Part **G** shows the predicted membrane localizations of these proteins. The sector letters A-F correspond to the diagram letters. The endoplasmic reticulum (ER) is continuous with perinuclear space (PNS). For simplicity, nuclear pores are not shown. Drawing is not to scale

**Fig. 2:**
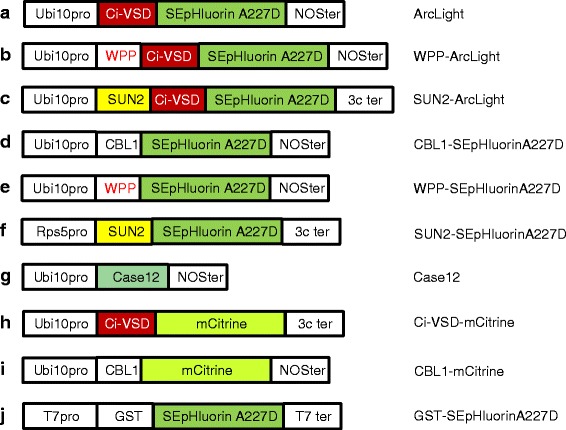
Constructs used in this study. The construct letters (**a-f**) correspond to the diagram letters in Fig. 1. SEpHluorinA227D, Ci-VSD and Case12 are defined in the text. The CBL1 motif is a 12 amino acid sequence from the CBL1 protein that contains a myristolated glycine and a palmitolated cysteine, which tether the fluorescent fusion protein to the cytoplasmic surface of the plasma membrane [25]. The WPP sequence, which contains a Trp(W)-Pro(P)-Pro motif that is highly conserved in all land plants [22], consists of amino acids 28–131 of *Arabidopsis* RANGAP1 and is sufficient for targeting fusion proteins to the outer nuclear membrane [23]. The SUN2 protein, which is 455 amino acids in length, has one transmembrane domain that can localize SUN2-fusion proteins at the inner nuclear membrane surface [44, 45] . In constructs (**a-f**) and (**g-i**), the gene encoding the fluorescence reporter is under the control of the ubiquitously-expressed Ubi10 plant promoter [39]. Construct F contains the root-specific Rps5 promoter [40]. Ci-VSD-mCitrine corresponds to VSFP3.1_mCitrine [28]. The constructs (**a-i**) contain either the nopaline synthase (NOS) or 3C transcriptional terminator. Construct (**j**) is designed for expression of GST-tagged SEpHluorinA227D in *E. coli* and contains the phage T7 promoter and terminator. The amino acid sequences of SEpHluorinA227D and environmentally-insensitive monomeric (m)Citrine compared to wild-type GFP and SEpHluorin are shown in Additional file 1: Figure S1. The constructs are not drawn to scale

Diagrams of ArcLight [11, 12] and various derivatives used in this study are depicted in Fig. 1a-f. The corresponding transgenic constructs introduced into *Arabidopsis* are shown in Fig. 2a-f. The predicted cellular locations of the fluorescent protein reporter with respect to the specific membrane targeting sequence are shown schematically in Fig. 1g.

The fluorescent proteins tested include: classic ArcLight (Fig. 1a), which - in the absence of any other membrane targeting sequence - is directed to the plasma membrane by the Ci-VSD (Fig. 1g, sector A); ArcLight joined at the N-terminus to the WPP domain of *Arabidopsis* RAN GTPASE ACTIVATING PROTEIN 1 (RANGAP1) (Fig. 1b) [22, 23], which promotes targeting to the outer nuclear membrane (Fig. 1g, sector B); and ArcLight fused at the N-terminus to the *Arabidopsis* SAD1/UNC-84 DOMAIN PROTEIN 2 (SUN2), which contains one transmembrane domain (Fig. 1c) and is able to target the protein to the inner nuclear membrane [24] (Fig. 1g, sector C).

In other constructs, we tested the importance of the transmembrane Ci-VSD in voltage-sensing by replacing it with either an *Arabidopsis* CALCINEURIN B-LIKE PROTEIN 1 (CBL1) plasma membrane targeting peptide [25] at the N-terminus (Fig. 1d), which situates the fluorescent reporter at the cytoplasmic surface of the plasma membrane (Fig. 1g, sector D); an N-terminal WPP domain (Fig. 1e), which places the fluorescent reporter at the cytoplasmic surface of the outer nuclear membrane (Fig. 1g, sector E); or an N-terminal fusion to inner nuclear membrane protein SUN2 (Fig. 1f), which positions the fluorescent reporter in the perinuclear space (Fig. 1g, sector F).

For comparative purposes, we used transgenic plants expressing the intensity-based free calcium concentration sensor Case12 (Calcium sensor 12) [26] (Fig. 2g) and mCitrine, which has been modified to reduce environmental sensitivity [27], joined to either Ci-VSD [28] (Fig. 2h) or CBL1 (Fig. 2i). A GST-tagged SEpHluorinA227D (Fig. 2j) was expressed in *E. coli* and isolated to test as a soluble variant of ArcLight. The amino acid sequences of wild-type GPF, mCitrine, SEpHluorin and SEpHluorinA227D are shown in Additional file 1: Figure S1.

**Fig. 3:**
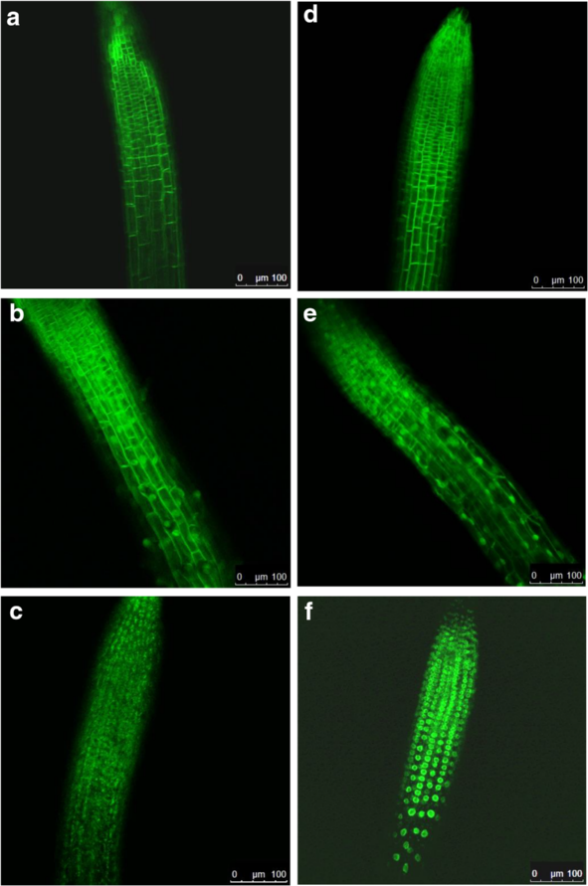
Fluorescent confocal images of transgenic plant roots expressing plasma membrane and nuclear membrane-localized GEVIs and derivatives. Images show the area of the root tip (meristem) and adjacent transition zone. The white bars on the bottom right indicate 100 μm. **a** ArcLight; **b** WPP-ArcLight; **c** SUN2-ArcLight; **d** CBL1-SEpHluorinA227D; **e** WPP-SEpHluorinA277D; **f** SUN2-SEpHluorinA277D. The letters correspond to those in the diagrams and constructs in Figs. 1 and 2, respectively

Transgenic *Arabidopsis* lines expressing ArcLight and various derivatives in root cells were produced and screened for strong and uniform expression levels of the transgene throughout the area of the root under investigation (typically the transition zone extending into the root apical meristem) as well as for specificity of membrane targeting and absence of visible aggregate formation. As anticipated, ArcLight (Fig. 1a) and CBL1-SEpHluorinA227D (Fig. 1d) were largely localized to the plasma membrane (Fig. 3a and d) with particularly distinct and bright plasma membrane fluorescence for CBL1-SEpHluorin, which lacks the Ci-VSD. The WPP fusion proteins (WPP-ArcLight and WPP-SEpHluorinA227D; Fig. 1b and e, respectively) were visualized at the nuclear periphery but plasma membrane localization was also observed (Fig. 3b and e, respectively), particularly for WPP-ArcLight, which contains the Ci-VSD. SUN2-SEpHluorinA227D, which lacks the Ci-VSD (Fig. 1f), localized almost exclusively at the nuclear rim (Fig. 3f) whereas SUN2-ArcLight, which contains the Ci-VSD (Fig. 1c), accumulated at both the plasma membrane and nuclear membrane and tended to aggregate (Fig. 3c). Thus, the dominance of the Ci-VSD as a plasma membrane-targeting motif reduced the preferential nuclear deposition of fluorescent reporters containing an additional nuclear membrane targeting signal and increased the possibility of fluorescent protein aggregation. Nuclear membrane targeting by SUN2 may be more specific than that achieved with WPP because the former involves a transmembrane domain whereas the latter is likely to associate more loosely with the membrane through electrostatic interactions.

Transgenic plants expressing Case12 displayed diffuse fluorescence that was particularly strong at the root tip whereas fluorescence was localized at the plasma membrane in root cells of transgenic plant expressing Ci-VSD-mCitrine and CBL1-mCitrine (Additional file 2: Figure S2). Expression of ArcLight and derivatives did not noticeably affect the phenotype of the transgenic plants, which grew and reproduced normally (data not shown).

### External ATP (eATP)

**Fig. 4:**
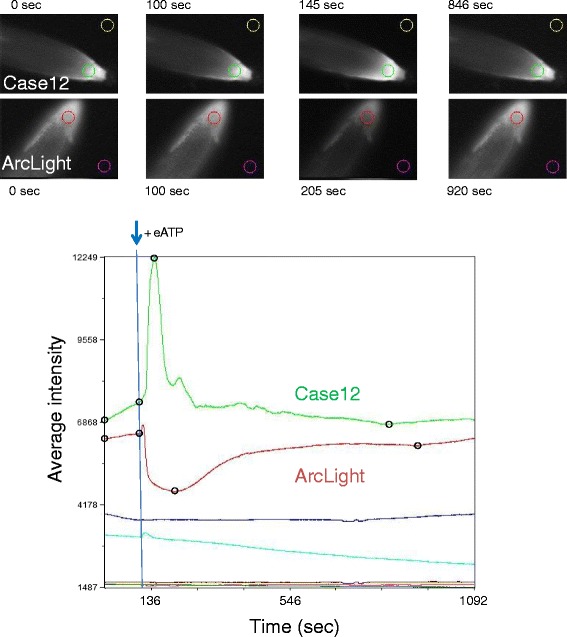
Comparison of Case12 and ArcLight responses to eATP. **Top:** MiCAM images of root tips of plants expressing ArcLight and Case12 with colored circles indicating the root and background regions used for the graphs. The images correspond to the beginning of the experiment (0 s), addition of ATP (100 s), highest response (145 s, Case12, increase of fluorescence; 205 s ArcLight, decrease of fluorescence) and recovery (846 s Case12; 920 s ArcLight), which can also be seen in the open black circles on the traces. **Bottom:** MiCAM raw data files were imported into Metamorph and combined into one stack for comparison of fluorescence intensity changes. The traces derived from the colored circled areas at the top are displayed over a time period of 1092 s. Either 2 mM ATP or buffer was added at approximately 100 sec as indicated by the blue arrow. The red and green traces represent the responses of ArcLight and Case12, respectively, to eATP addition. Pink and gold traces show the corresponding backgrounds for ArcLight and Case12, respectively. Turquoise and blue traces show the buffer controls for ArcLight and Case12, respectively. Dark red and dark green traces indicate background for buffer controls for ArcLight and Case12, respectively (MiCAM images not shown)

A previous study demonstrated that addition of 2 mM extracellular ATP (eATP) to roots of plants expressing a FRET-based calcium sensor elicited a large peak of fluorescence, indicative of increased intracellular free calcium, followed by oscillations and a gradual recovery to approach the baseline over a period of approximately 10 min [29]. We observed a similar response in root cells of transgenic seedlings expressing the fluorescence intensity-based free calcium sensor Case12 following the addition of 2 mM eATP (Fig. 4, Case12). The expected response of Case12 to eATP application validated our experimental system and provided a known signal that could be compared to the responses of ArcLight and derivatives to eATP treatments.

**Fig. 5:**
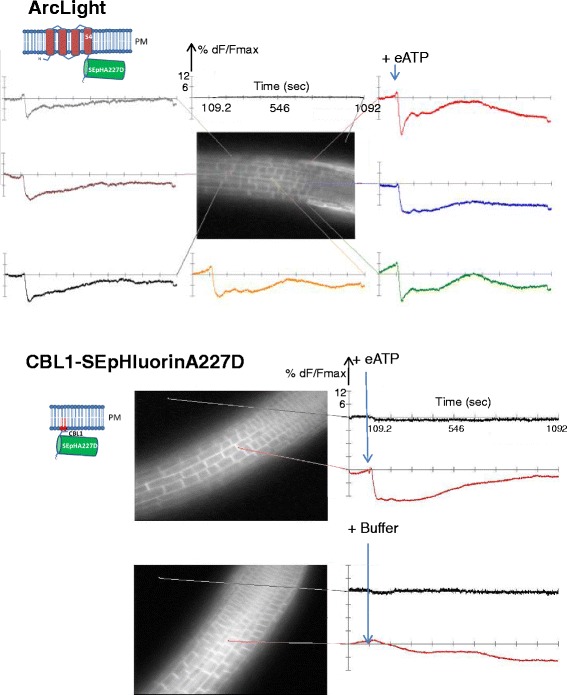
Similar responses of ArcLight and CBL1-SEpHluorinA227D to eATP. The traces derived from the regions of the root indicated by the connecting lines (MiCAM image at 0 s, 20x objective) are displayed over a time period of 1092 s. Either 2 mM ATP or buffer was added at approximately 100 s as indicated by the blue arrows. Fractional fluorescence changes (%dF/F_max_) were calculated by the BV-Analyzer software supplied with the MiCAM camera. The divisions of the Y-axis are set at 6 %. The X-axis shows time in seconds. **Top:** Responses of ArcLight to eATP addition are shown for multiple cells within the root. All cells show a qualitatively similar response. The background trace, which remains unchanged following addition of eATP, is shown above the MiCAM image. **Bottom:** Response of CBL1-SEpHluorinA227D to addition of eATP or buffer. The observed trace resembles that seen with ArcLight. The background trace is shown in black

ArcLight displayed a different response from Case12, with an initial small peak of fluorescence directly after eATP addition followed by a rapid decrease in fluorescence and gradual increase to approach the baseline (Fig. 4, ArcLight). The experimental setup allowed the observation of simultaneous changes in fluorescence intensity of ArcLight in multiple cells within the root (Fig. 5, top). Although the decrease in fluorescence intensity of ArcLight would be consistent with depolarization of the plasma membrane [11, 12], replacing the transmembrane segment Ci-VSD with the CBL1 membrane-tethering motif did not alter the response following exposure to eATP (Fig. 5, bottom). This indicates that the voltage sensitive domain Ci-VSD has no impact on the fluorescence response of ArcLight in plants. The dispensability of the voltage sensor suggests that ArcLight is not responding to voltage but to pH through its SEpHluorin base following eATP application.

**Fig. 6:**
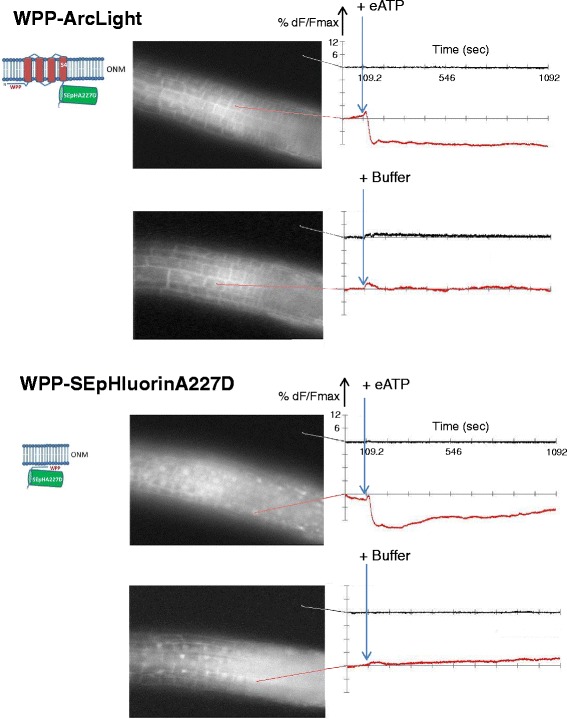
Responses of WPP-ArcLight and WPP-SEpHluorinA227D to eATP. Time period, display settings and sampling time are the same as for Fig. 5

**Fig. 7:**
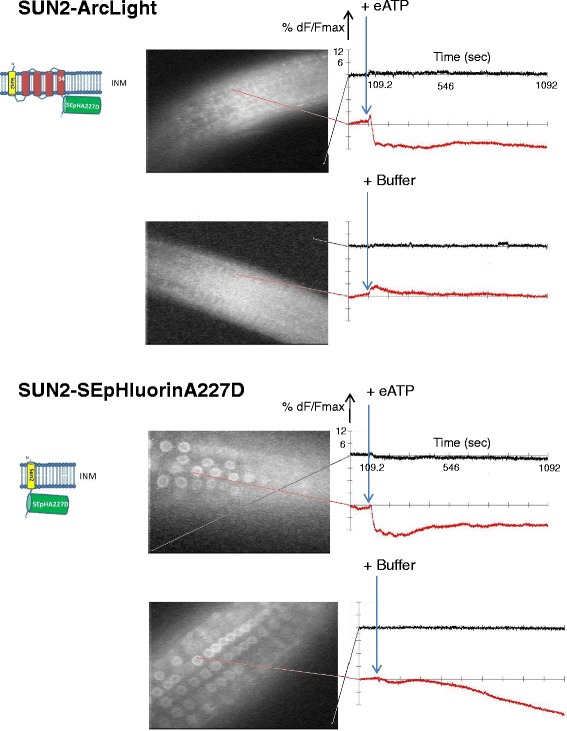
Responses of SUN2-ArcLight and SUN2-SEpHluorinA227D to eATP. Time period, display settings and sampling time are the same as for Fig. 5. The only difference is that for SUN2-SEpHluorinA277D (bottom) the MiCAM image was made using a 40x objective

Treatments with 2 mM eATP provoked similar reductions of fluorescence, irrespective of the presence or absence of the Ci-VSD, of the nuclear targeted proteins: WPP-ArcLight and WPP-SEpHluorinA227D (Fig. 6 top and bottom, respectively) and SUN2-ArcLight and SUN2-SEpHluorinA277D (Fig. 7 top and bottom, respectively). The latter result is noteworthy for monitoring changes specifically at a nuclear membrane given the virtually exclusive localization of the SUN2-SEpHluorinA227D at the nuclear rim (Fig. 3f and Fig. 7, bottom). Multiple cells or nuclei within roots displayed similar signals following addition of eATP in all transgenic lines tested (Additional file 3: Figure S3) indicating that the plasma membrane and nuclear membranes respond in a coordinated manner to eATP treatments.

All of the observed responses to eATP depended on the fluorescent proteins being in a cellular context because soluble GST-SEpHluorinA227D protein did not display any changes in fluorescence intensity when ATP was added to the solution (Additional file 4: Figure S4, top). In addition, negligible responses to eATP application were observed in plants expressing environmentally-insensitive mCitrine fused to either Ci-VSD or CBL1 (Additional file 5: Figure S5).

### ITMV and Light

To determine further effects on ArcLight fluorescence, we tested two additional stimuli that might be expected to provoke changes in membrane potential: induced transmembrane voltage (ITMV) [30, 31] and light [32, 33]. For ITMV experiments, seedlings were placed in a chamber flanked by two electrodes and subjected to an electric pulse of 2.5 V. For experiments using additional light, seedlings were placed in an agarose-pad-chamber and illuminated with various wavelengths of light in addition to continuous illumination at 500/20 nm, which is the excitation wavelength of ArcLight.

**Fig. 8:**
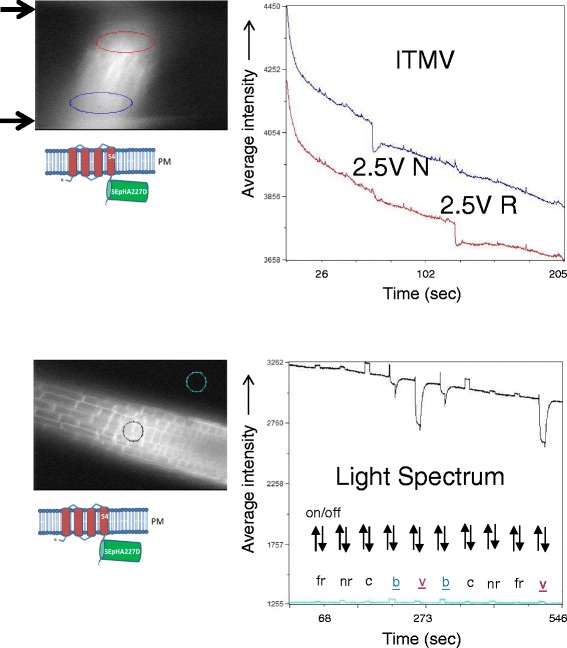
Responses of ArcLight to ITMV and additional illumination by different wavelengths of light. **Top** – induced transmembrane voltage (ITMV): Electrodes are positioned at the black arrows to the left of the MiCAM image. Root regions close to the electrodes that were used to make the graph are circled in red and blue to correspond to the cognate traces in the graph. Images were acquired at 200 ms intervals over a time period of 205 s. Voltage pulses (2.5 V with a duration of 200 ms) were applied at approximately 60 s and 120 s for normal (N) and reverse (R) polarities, respectively. ArcLight in the two regions responds in an opposite manner depending on the polarity of the pulse. The different effect in the two regions can be explained by the proximity of the responding cells to the depolarising electrode (i.e. cathode). With ‘normal polarity’ (stimulus at t = 60 s) the bottom electrode is the cathode and the blue circled cells responded by a cytoplasmic pH-drop, whereas with ‘reverse polarity’ (stimulus at t = 120 s) the top electrode is the cathode and the red circled cells responded. **Bottom** - additional illumination: Light spectrum details are provided in Methods section. Regions sampled are circled in the MiCAM image. Images were acquired at 100 ms intervals over a time period of 546 s. Duration of light pulses (on/off) was 10 s. Abbreviations: fr, far red; nr, near red; c, cyan; b, blue; v, violet. Under blue and violet illumination, ArcLight decreases in fluorescence intensity due to photobleaching, which is more pronounced when light of high energy (violet = 390 nm) is used as compared to lower energy (blue = 438 nm). The recovery of fluorescence after the bleaching light has been switched off is due to diffusion of unbleached fluorescent proteins into the focal plane of the imaging objective, an effect known as FRAP (Fluorescence recovery after photo bleaching). The small increases in the signal during illumination with far red, near red and cyan result from insufficient spectral separation of the illuminating light from the optical emission path of the microscope

Both ITMV and light in the blue and violet wavelengths elicited changes in fluorescence intensity of ArcLight in root cells (Fig. 8). However, similar changes in fluorescence were observed with soluble GST-pHluorinA227D (Additional file 4: Figure S4, middle and bottom), indicating that the responses – in contrast to those observed with eATP treatment - did not require the fluorescent reporter to be membrane-localized in a cellular context. Plasma membrane-anchored CBL1-SEpHluorinA227D displayed responses to blue and violet light resembling those observed with ArcLight (Additional file 6: Figure S6, top). However, the fluorescence of environmentally-insensitive mCitrine fused to either Ci-VSD or CBL1 in root cells remained largely unchanged under additional light illumination at all wavelengths (Additional file 6: Figure S6, middle and bottom), demonstrating that not all GFP-related fluorescent proteins respond in a similar manner to additional light.

## Discussion

Our study was designed to test the feasibility of using the fluorescence intensity-based GEVI ArcLight, which has been used as a voltage indicator in neurons, to monitor voltage changes at the plasma membrane and nuclear membranes in root cells. The membrane-associated fluorescent reporters were expressed well in *Arabidopsis* root cells. The voltage-sensing Ci-VSD conferred good targeting to the plasma membrane in the absence of additional targeting motifs. For reasons that are not completely clear, the Ci-VSD tended to promote protein aggregation and/or interfere with the specificity of nuclear envelope targeting when a nuclear membrane targeting sequence was also present.

As expected from previous work in neural cells, ArcLight and Ci-VSD-containing derivatives situated in these membrane systems responded robustly to eATP treatments by displaying transient reductions in fluorescence intensity. However, similar reductions in fluorescence intensity were observed with ArcLight derivatives lacking the voltage sensor Ci-VSD, indicating that the observed responses did not rely on voltage-sensing ability of the fluorescent protein. Therefore, decreased fluorescence intensity of ArcLight in response to eATP application in root cells is best interpreted as reflecting the pH sensitivity of its SEpHluorin base. In neurons, the pH sensitivity of ArcLight is less of a concern because H^+^-fluxes and pH changes during neuronal activity are of minor importance. By contrast, H^+^-ions contribute substantially to depolarisation and electrical activities in plants [34].

The decrease of ArcLight fluorescence in response to pH changes following eATP treatment can be understood as follows: The transient depolarization induced by eATP is accompanied by a large increase in free cytoplasmic calcium ion concentration ([Ca2+]cyt), as shown by the transient increase in fluorescence of Case12. Both depolarisation and [Ca2+]cyt transient are the result of cation channel activities, which are mainly K^+^-channels, but these are rather nonspecific and can also conduct H^+^ ions. Since there is a membrane potential (negative inside the cell with respect to the outside) and a pH-gradient between the outer medium and cytoplasm (the pH of the apoplast is normally between 4.5 and 6.5 [35], whereas cytoplasmic pH is usually around 7.3 [36]), protons run down their electrochemical gradient upon cation channel opening, enter the cell, and acidify its internal contents. The SEpHluorin component of ArcLight responds to H^+^-ion plumes near the membrane and to cytoplasmic acidification, resulting in reductions in fluorescence. In this scenario, ArcLight responds primarily to the downstream consequence of a membrane voltage change (decreased pH) and not directly to the voltage change itself.

## Conclusions

In summary, although ArcLight and the derivatives tested here do not provide a direct sensor for voltage changes in plants, they can potentially be used as fluorescence intensity-based, membrane-localized indicators of pH changes at the cell surface and nuclear periphery. These fluorescence intensity-based pH indicators display robust responses and, following further validation and calibration, may provide facile alternatives to ratiometric-based pH indicators based on GFP [37]. The development of monochromatic GEVIs for use in plant systems will require the identification of fluorescent reporter proteins that are less sensitive than ArcLight to changes in pH.

## Methods

### Transgenic plants expressing fluorescent protein reporters

The nucleotide sequence of ArcLight, codon-optimized for expression in *Drosophila melanogaster* [38], was obtained from Dr. Michael Nitabach (Yale University) and then codon-optimized by our lab for *Arabidopsis* and synthesized by GeneScript. Transgenic constructs (Fig. 2) were produced using standard molecular biology techniques. The transgenes plus promoter (either Ubi10 [39] or Rps5 [40]) were assembled on modified pBC plasmids (Stratagene, Cat. Nr. 212215) between *Sal*1 and *Xho*I sites, and the entire transgene was inserted into the *Sal*1 site of binary vector pZP221 [41]. The respective binary vectors containing each transgene construct were introduced via *Agrobacterium*-mediated transformation into *Arabidopsis thaliana* ecotype Columbia-0 using the floral dip method [42]. All transgenic lines used in this study were generated in our lab. The Ubi10 promoter drives expression in the whole root, including root hairs. The RPS5 promoter is expressed primarily in the division zone-transition zone. The data shown are from lines that showed the best expression.

Seeds of transgenic plants were surface sterilized in 1.5 ml Eppendorf tubes by shaking them for 20 min in 1 ml of 70 % ethanol solution containing Triton X-100 (50 μl per 100 ml 70 % ethanol). The seeds were centrifuged in an Eppendorf centrifuge for 1 min, the supernatant removed, and the seeds were resuspended in 1 ml of 100 % ethanol and immediately pipetted onto a filter paper disk in a sterile hood. After air-drying for 1 h, seeds were sprinkled onto sterile, solid Murashige and Skoog (MS) medium in petri dishes, stratified by storing the plate at 4 °C for 3 days, and then transferred to a light incubator (23 °C, 16 h light, 8 h dark) for about one week of growth before being used for experiments as described.

### Expression of SEpHluorinA277D in E. coli

We expressed GST-tagged SEpHluorinA277D in *E. coli* strain BL21 (NEB, USA) using the expression vector pET-42a(+), which contains a GST-tag and multiple cloning site (Novagen, USA). The SEpHluorinA227D sequence was amplified using PCR as a *Bam*HI/*Hin*dIII fragment and cloned in frame into pET-42a(+) and introduced into BL21 cells. Production of GST-tagged SEpHluorinA227D protein was induced with isopropyl β-D-1-thiogalactopyranoside (IPTG) on agar plates overnight. The GST-tagged SEpHluorinA277D was isolated using the BugBuster GST-Bind Purification Kit (Novagen 79794–3 REF) and purified using the small scale batch method according to the manufacturer’s instructions.

### Confocal microscopy

Confocal images of seedlings growing on sterile, solid MS medium in petri dishes were acquired using the Leica TCS LSI microscope equipped with a 5x Z16 APO A zoom system (Leica Microsystems CMS GmbH, Germany, purchased from Major Instruments, Taiwan).

### Fluorescence imaging and data processing

Fluorescence changes were recorded with a fast CCD imaging system, MiCAM02-HR, which is specialized for both calcium ion and membrane voltage imaging applications (Brainvision Inc., Japan, purchased from Major Instruments, Taiwan), mounted on an Axiovert25 inverted fluorescence microscope (Carl Zeiss GmbH, Germany) equipped with 5x/0.12 (CP-Acromat), 20x/0.8 (Plan-Apochromat) and 40x/0.9 Pol (EC Plan-Neofluar) objectives and either an ET YFP filter cube (Ex ET 500/20, beam splitter T515p, Em ET 535/30) or an FITC filter cube (Ex HQ 480/40x, beam splitter Q 505 LPe, Em HQ 535/50 m). The light source for the microscope was a xenon short arc lamp without reflector, model XBO 150 W/CR OFR (OSRAM GmbH, Germany) housed in an OptoSource illuminator (Cairn Research Ltd., U.K. purchased via Major Instruments, Taiwan). Displays of results were obtained using Metamorph (Meta Imaging Series Software, Molecular Devices, USA) (Figs. 4, 8; Additional file 6: Figure S6) or data analysis software BV_Analyzer (ver1312) (Brainvision Inc., Japan) (Figs. 5, 6, 7; Additional file 3: Figure S3; Additional file 4: Figure S4; Additional file 5: Figure S5). In the latter case, the fractional fluorescence changes (dF/F_max_) shown in the figures were calculated using the processing function of BV_Analyzer. All experiments were performed multiple times and representative results are shown. After seedlings recovered from the experiments, they could be transferred to soil, where they grew and reproduced normally.

### External ATP (eATP)

For addition of eATP to seedlings, we used a microscope slide-sized, open-top bath chamber (a gift from Dr. Kai Konrad, University of Würzburg, Germany) made of 3 mm thick plexiglass (7.6 × 2.5 cm) with an oval, bevelled indentation (4.5 × 1.8 cm) and a large cover slip (6 × 2.5 cm) glued on the bottom. This chamber was filled with 1 % agarose in 1x imaging solution [5 mM potassium chloride, 10 mM MES hydrate, 10 mM calcium chloride, adjusted to pH5.8 with Tris(hydroxymethyl)aminomethane] [29, 43] to make tight fitting agar blocks. After solidification at room temperature, the agar block was removed and transferred to a round petri dish, where it was kept in 1x imaging solution until use, when it was cut into approximately 1 cm broad slices.

To mount an *Arabidopsis* seedling (1–2 weeks old) for fluorescence imaging, 400 μl 1x imaging solution was pipetted into the open-top chamber and an intact seedling removed from MS medium was positioned lengthwise into the imaging solution. A 1 cm slice of the agar block (as prepared above) was then placed on top of the area of the extended root to be imaged, leaving the root tip protruding on one side and the leaves on the other side. With fine forceps, the seedling was pulled carefully until the root tip was just under the agar block. Excess solution around the agar block was removed. The chamber was then mounted on the inverted microscope. A silicon tube was attached on one end to a Gilson Pipetman and - after filling the tube with 50 μl 2 mM ATP in 1x imaging solution or buffer for buffer-only control experiments as indicated in the figures - the other end was attached to a holder that is positioned just above the edge of the agar block under which the root has been positioned (Additional file 7: Figure S7A). Fluorescence recording was then started and the eATP solution was pipetted into the bath chamber at a specified time.

To test soluble GST-SEpHluorinA227D, 200 μl of the isolated protein in elution buffer EB (BugBuster GST-Bind Purification Kit, Novagen) (protein concentration approximately 30 μg/ml) was placed in the Bügelkammer (see section on ITMV) and 50 μl eATP solution, which also contained 30 μg/ml GST-tagged SEpHluorin to maintain the protein concentration, was added from a silicon tube positioned just above the edge of the cover slip.

### ITMV (induced transmembrane voltage)

For application of voltage pulses, we used a Grass SD stimulator (Grass, USA) connected to two electrodes mounted on a slide holder (Bügelkammer, Krüss GmbH, Germany). The two electrodes of the Bügelkammer are mounted on separate plastic stirrups and can be clamped down individually over the sample, thus positioning the electrodes at a distance of 200 μm. A 24 × 40 mm cover slip is placed in the open Bügelkammer, 200 μl 1x imaging solution [29, 43] is placed on the cover slip, and the first electrode is clamped down on the cover slip. A seedling is placed horizontally in the imaging solution above the first electrode and the second electrode is clamped down onto the cover slip such that the root is under the second electrode. An 18x18 mm cover slip is then placed on top of the root for stabilization (Additional file 7: Figure S7B). The area of the root between the two electrodes was imaged (see for example, Fig. 8a). To test soluble GST-SEpHluorinA227D, the isolated protein in elution buffer EB (BugBuster GST-Bind Purification Kit, Novagen) (protein concentration approximately 30 μg/ml) was placed in the Bügelkammer and covered with an 18x18 mm cover slip.

### Light treatment

To test the influence of additional light pulses on fluorescence intensity of ArcLight and derivatives (during continuous illumination at 500/20 nm, the excitation wavelength of ArcLight), a SPECTRA X Light Engine (Lumencor, USA, purchased from Major Instruments, Taiwan) was used for illumination at 390/18 nm (violet), 438/24 nm (blue), 475/28 nm (cyan), 589/15 nm (near red) and 632/22 nm (far red). The additional light at these wavelengths was shone at an angle of 30° and from a distance of 10 cm on a seedling mounted under an agar block in the open-top chamber as described for eATP experiments. The light intensity was set at 10 % on the SPECTRA X Light Engine and focused on the imaging plane. Automated turning on and off at certain wavelengths of the SPECTRA X Light Engine and at specific time points was achieved by using GhostMouse software (http://tw.vrbrothers.com) or was performed by hand. To test soluble GST-SEpHluorinA227D, the isolated protein in elution buffer EB (BugBuster GST-Bind Purification Kit, Novagen) (protein concentration approximately 30 μg/ml) was placed in the Bügelkammer, covered with a 18x18 mm cover slip, and illuminated with the SPECTRA X Light Engine as described above.

## Additional files

Additional file 1: Figure S1. Comparison of amino acid sequences of mCitrine, wild-type GFP, SEpHlourinA227 and SEpHluorin A227D. (PDF 62 kb) Additional file 2: Figure S2. Fluorescent confocal images of roots of transgenic plants expressing soluble Case12 and plasma membrane-localized Ci-VSD-mCitrine and CBL1-mCitrine. (PDF 610 kb) Additional file 3: Figure S3. Simultaneous changes in fluorescence intensity of reporter proteins in multiple root cells following eATP addition. (PDF 371 kb) Additional file 4: Figure S4. Responses of soluble GST-SEpHlorinA227D to eATP, light and ITMV. (PDF 197 kb) Additional file 5: Figure S5. Responses to Ci-VSD-Citrine and CBL1-Citrine to eATP. (PDF 225 kb) Additional file 6: Figure S6. CBL1-SEpHluorinA227D but not environmentally-insensitive mCitrine responds to additional illumination by different wavelengths of light. (PDF 627 kb) Additional file 7: Figure S7. Photographs showing mounted seedlings for treatments with eATP, light and ITMV. (PDF 196 kb)

## Abbreviations

ArcLight: SEpHluorinA227D fused to Ci-VSD
Case12: Calcium sensor 12
CBL1: CALCINEURIN B-LIKE PROTEIN 1. The *Arabidopsis* CBL1 protein (At4g17615) contains a myristolated glycine and a palmitolated cysteine, which tether the fluorescent fusion protein to the cytoplasmic surface of the plasma membrane
Ci-VSD: voltage sensing domain of *Ciona intestinalis* voltage-sensing phosphatase
eATP: extracellular ATP
FRET: Förster resonance energy transfer
GEVI: genetically-encoded voltage indicator
GST: glutathione S-transferase
ITMV: induced transmembrane voltage
mCitrine: monomeric citrine
RANGAP1: RAN GTPASE ACTIVATING PROTEIN 1
SEpHluorin: super-ecliptic(SE) pHluorin (**pH**-sensitive f**luor**escent prote**in**)
SEpHluorinA227D: super-ecliptic(SE) pHluorin (**pH**-sensitive f**luor**escent prote**in**) containing an A227D substitution that confers voltage sensitivity in neurons
SUN2: SAD1/UNC-84 DOMAIN PROTEIN 2. The *Arabidopsis* SUN2 protein (At3g10730) has one transmembrane domain that can localize SUN2-fusion proteins at the inner nuclear membrane surface
WPP: The WPP sequence contains a Trp(W)-Pro(P)-Pro motif consists of amino acids 28–131 of *Arabidopsis* RANGAP1 (At3g63130) that is sufficient for targeting fusion proteins to the outer nuclear membrane
