# Supplementary material for: Expression and testing in plants of ArcLight, a genetically–encoded voltage indicator used in neuroscience research
Source: BMC Plant Biol. 2015 Oct 12;15:245. doi: 10.1186/s12870-015-0633-z (PMC4603945; doi:10.1186/s12870-015-0633-z)
Supplement: Additional file 1: Figure S1. — Comparison of amino acid sequences of mCitrine, wild-type GFP, SEpHlourinA227 and SEpHluorin A227D. (PDF 62 kb) [file 12870_2015_633_MOESM1_ESM.pdf]

```
mcitrine    MVSKGEELFTGVVPILVELDGDVNGHKFSVSGEGEGDATY    40
wt_gfp      .MSKGEELFTGVVPILVELDGDVNGHKFSVSGEGEGDATY    39
sephl_a     .MSKGEELFTGVVPILVELDGDVNGHKFSVSGEGEGDATY    39
sephl_d     .MSKGEELFTGVVPILVELDGDVNGHKFSVSGEGEGDATY    39

mcitrine    GKLTLKFICTTGKLPVPWPTLVTTFGYGLMCFARYPDHMK    80
wt_gfp      GKLTLKFICTTGKLPVPWPTLVTTFSYGVQCFSRYPDHMK    79
sephl_a     GKLTLKFICTTGKLPVPWPTLVTTLTYGVQCFSRYPDHMK    79
sephl_d     GKLTLKFICTTGKLPVPWPTLVTTLTYGVQCFSRYPDHMK    79

mcitrine    QHDFFKSAMPEGYVQERTIFFKDDGNYKTRAEVKFEGDTL   120
wt_gfp      QHDFFKSAMPEGYVQERTIFFKDDGNYKTRAEVKFEGDTL   119
sephl_a     RHDFFKSAMPEGYVQERTIFFKDDGNYKTRAEVKFEGDTL   119
sephl_d     RHDFFKSAMPEGYVQERTIFFKDDGNYKTRAEVKFEGDTL   119

mcitrine    VNRIELKGIDFKEDGNILGHKLEYNYNSHNVYIMADKQKN   160
wt_gfp      VNRIELKGIDFKEDGNILGHKLEYNYNSHNVYIMADKQKN   159
sephl_a     VNRIELKGIDFKEDGNILGHKLEYNYNDHQVYIMADKQKN   159
sephl_d     VNRIELKGIDFKEDGNILGHKLEYNYNDHQVYIMADKQKN   159

mcitrine    GIKVNFKIRHNIEDGSVQLADHYQQNTPIGDGPVLLPDNH   200
wt_gfp      GIKVNFKIRHNIEDGSVQLADHYQQNTPIGDGPVLLPDNH   199
sephl_a     GIKANFKIRHNIEDGGVQLADHYQQNTPIGDGPVLLPDNH   199
sephl_d     GIKANFKIRHNIEDGGVQLADHYQQNTPIGDGPVLLPDNH   199

mcitrine    YLSYQSKLSKDPNEKRDHMVLLEFVTAAGITLGMDELYK    240
wt_gfp      YLSTQSALSKDPNEKRDHMVLLEFVTAAGITHGMDELYK    239
sephl_a     YLFTTSTLSKDPNEKRDHMVLLEFVTAAGITHGMDELYK    239
sephl_d     YLFTTSTLSKDPNEKRDHMVLLEFVTADGITHGMDELYK    239
```

mCitrine: environmentally insensitive
Wild-type GFP
SEpHluorin: pH sensitive
SEpHluorinA227D: voltage sensitive

F64L, S65T
"Super Ecliptic" (in SEpHluorin): increases fluorescence and red shifts the exitation peak from 475 to 491 nm

S147D, N149Q

confer pH sensitivity in pHluorin

S202F, Q204T, A206T

A227D confers voltage sensitivity in ArcLight

Figure S1, Matzke et al.

Figure S1: Comparison of amino acid sequences of mCitrine, wild-type GFP, SEpHlourinA227 and SEpHluorinA227D

Various amino acid substitutions have been made to wild-type (WT) GFP to produce environmentally-sensitive fluorescent proteins. The F64L and S65T substitutions were made to produce Super-Ecliptic (SE)pHluorin (pH-sensitive fluorescent protein) [1]. The substitutions that confer pH sensitivity include S147D, N149Q, S202F, Q204T, A206T [2]. The A227D substitution confers voltage-sensitivity to ArcLight in neurons [3]. Modifications to reduce environmental sensitivity of mCitrine are described in reference [4].

References

1. Miesenbock G: **Synapto-pHluorins: genetically encoded reporters of synaptic transmission.** Cold Spring Harb Protoc 2012, **2012**:213-217.

2. Miesenböck G, De Angelis DA, Rothman JE: **Visualizing secretion and synaptic transmission with pH-sensitive green fluorescent proteins.** *Nature* 1998, **394**:192-195.

3. Jin L, Han Z, Platisa J, Wooltorton JR, Cohen LB, Pieribone VA: **Single action potentials and subthreshold electrical events imaged in neurons with a fluorescent protein voltage probe.** *Neuron* 2012, **75**:779-785.

4. Griesbeck O, Baird GS, Campbell RE, Zacharias DA, Tsien RY: **Reducing the environmental sensitivity of yellow fluorescent protein. Mechanism and applications.** *J Biol Chem* 2001; **276**:29188-29194.
