# Supplementary material for: Expression and testing in plants of ArcLight, a genetically–encoded voltage indicator used in neuroscience research
Source: BMC Plant Biol. 2015 Oct 12;15:245. doi: 10.1186/s12870-015-0633-z (PMC4603945; doi:10.1186/s12870-015-0633-z)
Supplement: Additional file 2: Figure S2. — Fluorescent confocal images of roots of transgenic plants expressing soluble Case12 and plasma membrane-localized Ci-VSD-mCitrine and CBL1-mCitrine. (PDF 610 kb) [file 12870_2015_633_MOESM2_ESM.pdf]

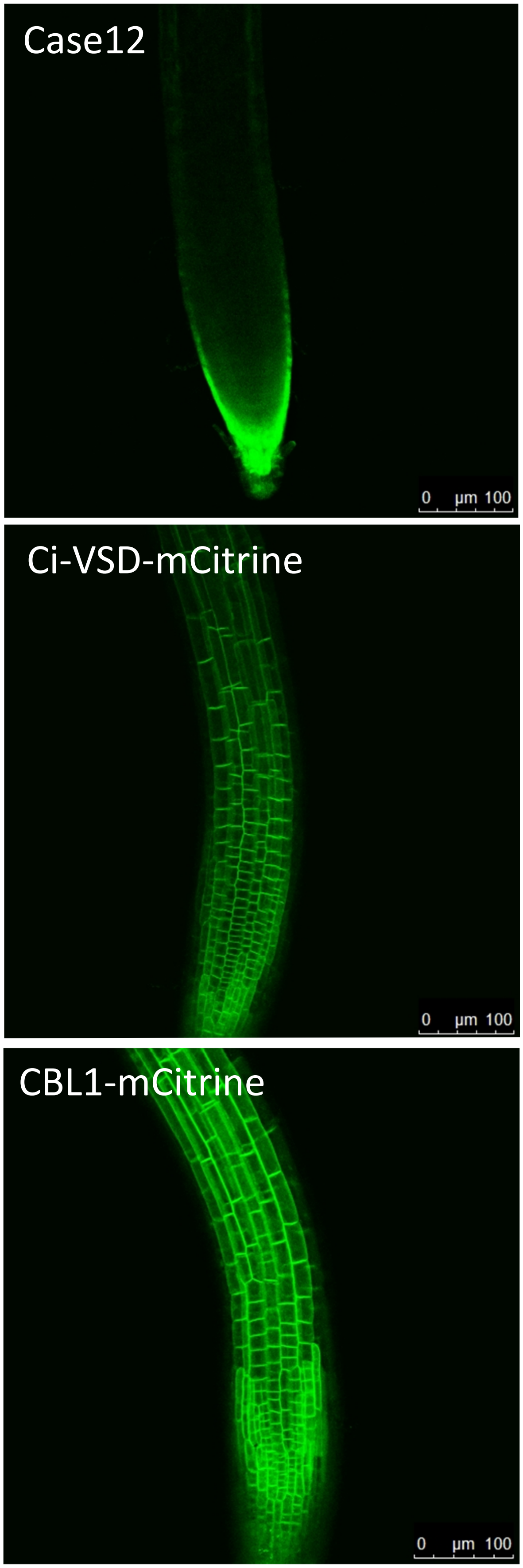

Figure S2, Matzke et al.

Figure S2: Fluorescent confocal images of roots of transgenic plants expressing soluble Case12 and plasma membrane-localized Ci-VSD-mCitrine and CBL1-mCitrine

Images show the area of the root tip (meristem) and adjacent transition zone. The white bars on the bottom right indicate 100µm. The corresponding constructs are in **Figure 2.**
