# Supplementary material for: Expression and testing in plants of ArcLight, a genetically–encoded voltage indicator used in neuroscience research
Source: BMC Plant Biol. 2015 Oct 12;15:245. doi: 10.1186/s12870-015-0633-z (PMC4603945; doi:10.1186/s12870-015-0633-z)
Supplement: Additional file 3: Figure S3. — Simultaneous changes in fluorescence intensity of reporter proteins in multiple root cells following eATP addition. (PDF 371 kb) [file 12870_2015_633_MOESM3_ESM.pdf]

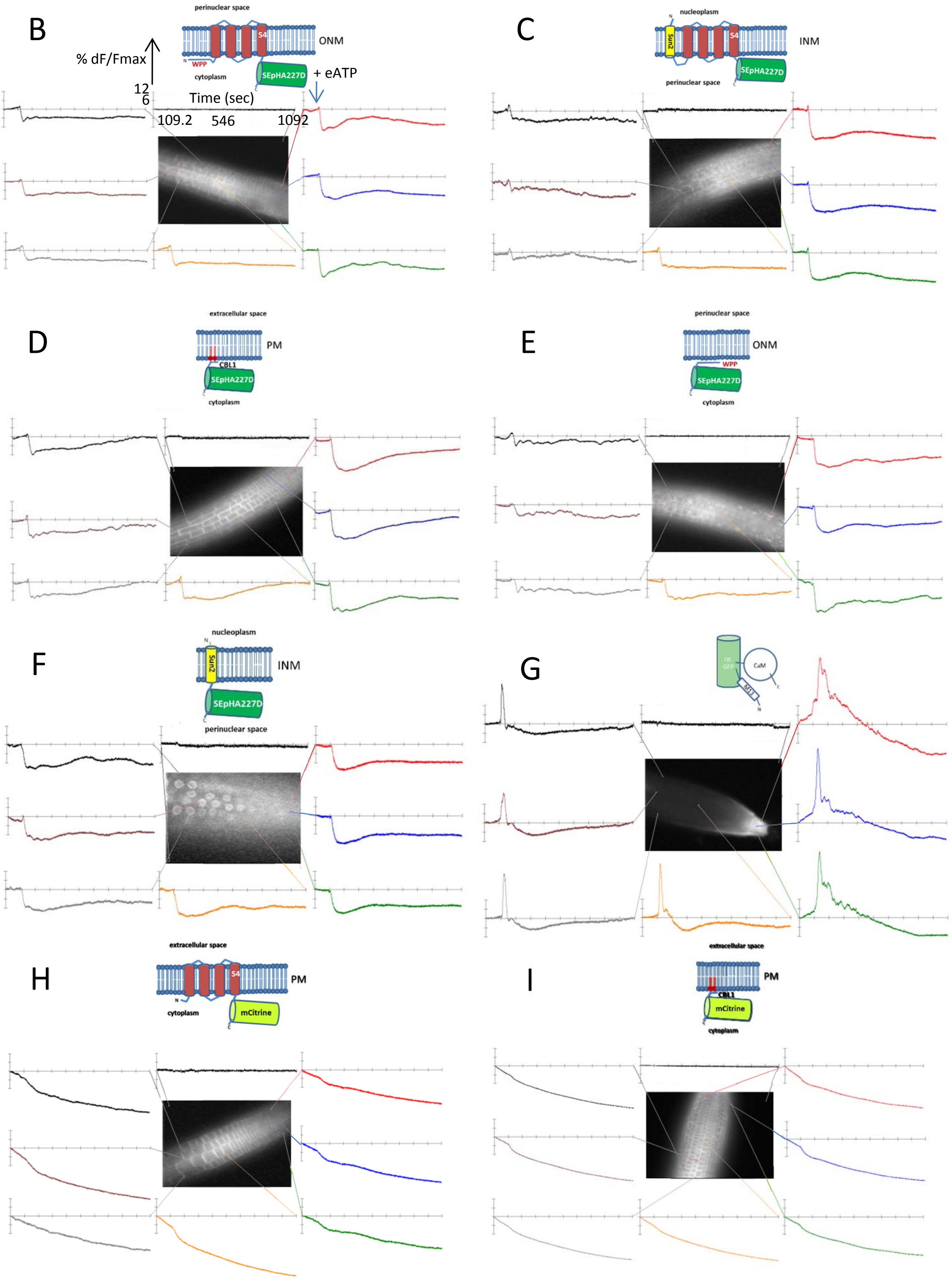

Figure S3, Matzke et al.

Figure S3: Simultaneous changes in fluorescence intensity of reporter proteins in multiple root cells following eATP addition

The traces derived from the regions of the root indicated by the connecting lines (MiCAM image at 0sec, 20x objective except in F, which used a 40x objective) are displayed over a time period of 1092 sec. The 2mM ATP was added at approximately 100 sec indicated by the blue arrow in part B. Fractional fluorescence changes (%dF/F_{max}) were calculated by the BV-Analyzer software supplied with the MiCAM camera. The divisions of the Y-axis are set at 6%. The X-axis shows time in seconds.

(B) WPP-ArcLight; (C) SUN2-ArcLight; (D) CBL1-SEpHluorinA277D; (E) WPP-SEpHluorinA277D; (F) SUN2-SEpHluorinA277D; (G) Case12; (H) Ci-VSD-mCitrine; (I) CBL1-mCitrine. The letters correspond to the constructs shown in **Figure 2** (Construct A, Arclight, is shown in **Figure 5**). For any given fluorescent reporter, all cells show a qualitatively similar response. Background traces are shown above the MiCAM images and stay flat following addition of eATP.
