# Supplementary material for: Expression and testing in plants of ArcLight, a genetically–encoded voltage indicator used in neuroscience research
Source: BMC Plant Biol. 2015 Oct 12;15:245. doi: 10.1186/s12870-015-0633-z (PMC4603945; doi:10.1186/s12870-015-0633-z)
Supplement: Additional file 4: Figure S4. — Responses of soluble GST-SEpHlorinA227D to eATP, light and ITMV. (PDF 197 kb) [file 12870_2015_633_MOESM4_ESM.pdf]

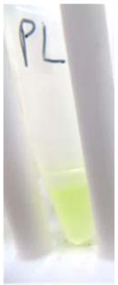

Purified GST-tagged SEpHluorinA227D
made in *E. coli*

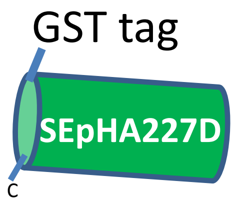

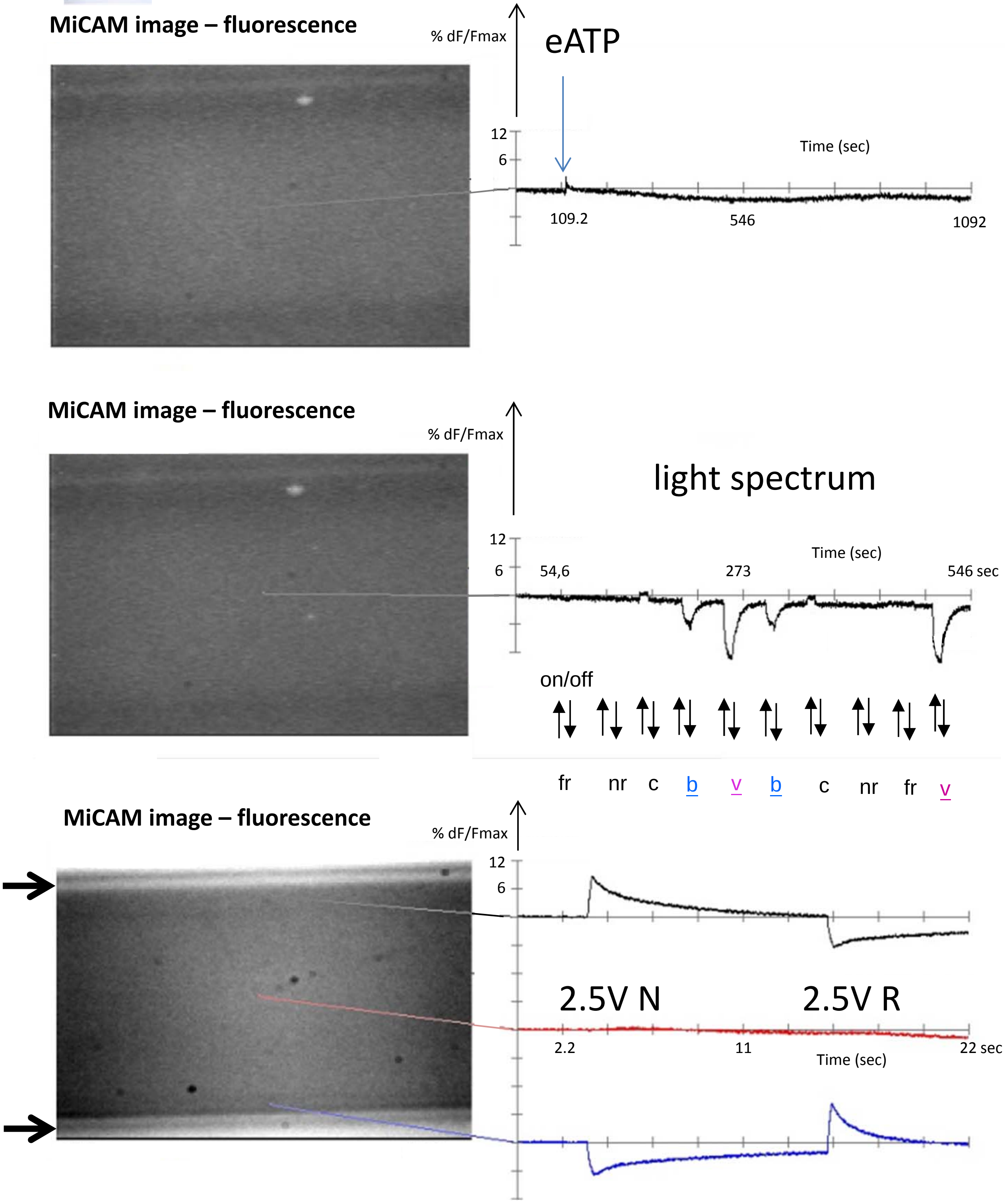

Figure S4, Matzke et al.

Figure S4: **Responses of soluble GST-SEpHlorinA227D to eATP, light and ITMV**

The concentration of GST-SE-pHluorin was approximately 30µg/ml. Details of experiments are supplied in the Methods section. Fractional fluorescence changes (%dF/F_{max}) were calculated by the BV-Analyzer software supplied with the MiCAM camera. The divisions of the Y-axis are set at 6%. The X-axis shows time in seconds.

Top- eATP addition: The trace is derived from measuring the fluorescence in the center of the solution (MiCAM image at 0sec, 20x objective) over a time period of 1092sec. Fifty microliters of a 2mM solution ATP were added at approximately 100sec as indicated by the blue arrow. Unlike membrane-localized ArcLight and derivatives in roots, soluble GST-SEpHluorinA277D does not change fluorescence intensity following addition of eATP.

Middle – additional illumination: Light spectrum details are provided in the Methods section. The region sampled is pinpointed in the center of the MiCAM image of the solution. Images were acquired at 100ms intervals over a time period of 546sec. Duration of light pulses (on/off) was 10sec. Abbreviations: fr, far red; nr, near red; c, cyan; b, blue; v, violet. Similarly to ArcLight in roots, soluble GST-SEpHluorin decreases in fluorescence under blue and violet illumination due to photobleaching and FRAP (see **Figure 8**).

Bottom – ITMV: Electrodes are positioned at the black arrows to the left of the MiCAM image. Regions of the solution at the center and close to the two electrodes that were used to make the graphs are pinpointed in red, black and blue to correspond to the cognate traces in the graph. Images were acquired at 4ms intervals over a time period of 22sec. Voltage pulses (2.5V with a duration of 200ms) were applied at approximately 3sec and 13sec for normal (N) and reverse (R) polarities, respectively. In the regions close to one of the two electrodes, soluble GST-SEpHluorin responds in an opposite manner (increase or decrease in fluorescence) depending on

the polarity of the pulse. In the center, no change in fluorescence was observed. These results are a consequence of the electrolysis of water, which can be understood as follows. The purified protein is dissolved in a salt containing buffer. When a voltage is applied in this buffer then electrolysis occurs. This means that protons are produced at the anode and are consumed at the cathode as long as a voltage is set. Consequently, the proximate environment of the anode acidifies while the environment of the cathode becomes alkaline. This is reported in this experiment by the purified SEpHluorin. The fluorescence is quenched by H+ near the anode and in parallel the fluorescence increases near the cathode due to alkalization. The data report pH-shifts in the milieu but do not provide evidence for a voltage sensitivity of the reporter protein.
