# Supplementary material for: Expression and testing in plants of ArcLight, a genetically–encoded voltage indicator used in neuroscience research
Source: BMC Plant Biol. 2015 Oct 12;15:245. doi: 10.1186/s12870-015-0633-z (PMC4603945; doi:10.1186/s12870-015-0633-z)
Supplement: Additional file 5: Figure S5. — Responses to Ci-VSD-Citrine and CBL1-Citrine to eATP. (PDF 225 kb) [file 12870_2015_633_MOESM5_ESM.pdf]

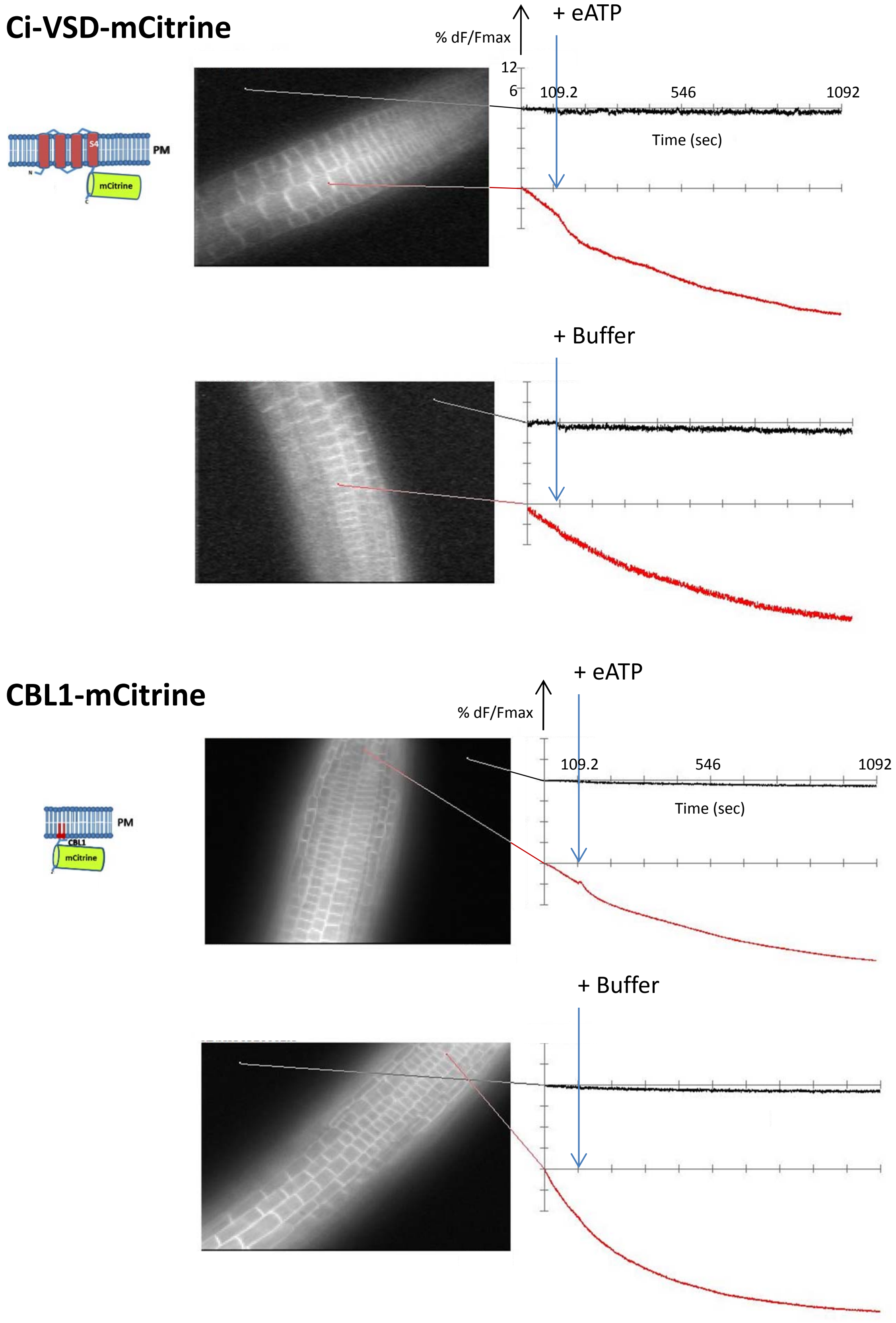

Figure S5, Matzke et al.

Figure S5: **Responses to Ci-VSD-Citrine and CBL1-Citrine to eATP**

The traces derived from the regions of the root indicated by the connecting lines (MiCAM image at 0sec, 20x objective) are displayed over a time period of 1092sec. Either 2mM ATP or buffer was added at approximately 100sec as indicated by the blue arrows. Fractional fluorescence changes (%dF/F_{max}) were calculated by the BV-Analyzer software supplied with the MiCAM camera. The divisions of the Y-axis are set at 6%. The X-axis shows time in seconds. Although gradual bleaching is observed, also in the buffer control, negligible responses to eATP application were observed in plants expressing environmentally-insensitive mCitrine fused to either Ci-VSD or CBL1.
