# Supplementary material for: Expression and testing in plants of ArcLight, a genetically–encoded voltage indicator used in neuroscience research
Source: BMC Plant Biol. 2015 Oct 12;15:245. doi: 10.1186/s12870-015-0633-z (PMC4603945; doi:10.1186/s12870-015-0633-z)
Supplement: Additional file 6: Figure S6. — CBL1-SEpHluorinA227D but not environmentally-insensitive mCitrine responds to additional illumination by different wavelengths of light. (PDF 627 kb) [file 12870_2015_633_MOESM6_ESM.pdf]

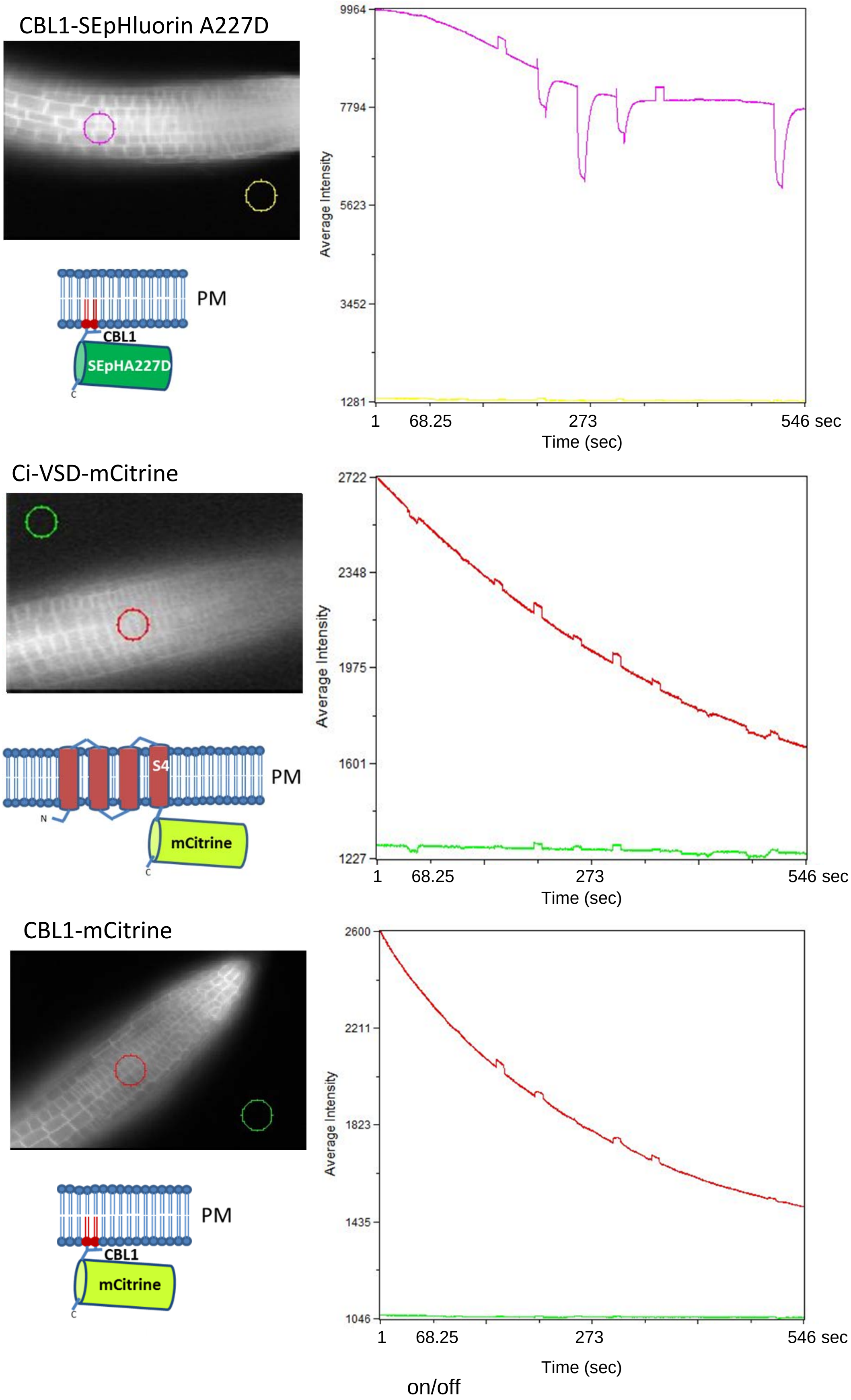

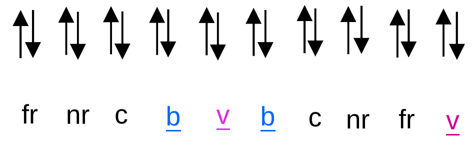

Figure S6, Matzke et al.

Figure S6: CBL1-SEpHluorinA227D but not environmentally-insensitive mCitrine responds to additional illumination by different wavelengths of light

Light spectrum details are provided in the Methods section. Regions sampled are circled in the MiCAM images. Images were acquired at 100ms intervals over a time period of 546sec. Duration of light pulses (on/off) was 10sec. Abbreviations: fr, far red; nr, near red; c, cyan; b, blue; v, violet. Under blue and violet illumination, CBL1-SEpHluorin (**top**), like ArcLight decreases in fluorescence intensity due to photobleaching and FRAP (see **Figure 8**). By contrast, the mCitrine derivatives (**middle and bottom**) do not show similar decreases in fluorescence because citrine is photobleaching-insensitive. The small increases in the signal during illumination with far red, near red and cyan result from insufficient spectral separation of the illuminating light from the optical emission path of the microscope.

.
