# Supplementary material for: Expression and testing in plants of ArcLight, a genetically–encoded voltage indicator used in neuroscience research
Source: BMC Plant Biol. 2015 Oct 12;15:245. doi: 10.1186/s12870-015-0633-z (PMC4603945; doi:10.1186/s12870-015-0633-z)
Supplement: Additional file 7: Figure S7. — Photographs showing mounted seedlings for treatments with eATP, light and ITMV. (PDF 196 kb) [file 12870_2015_633_MOESM7_ESM.pdf]

A

B

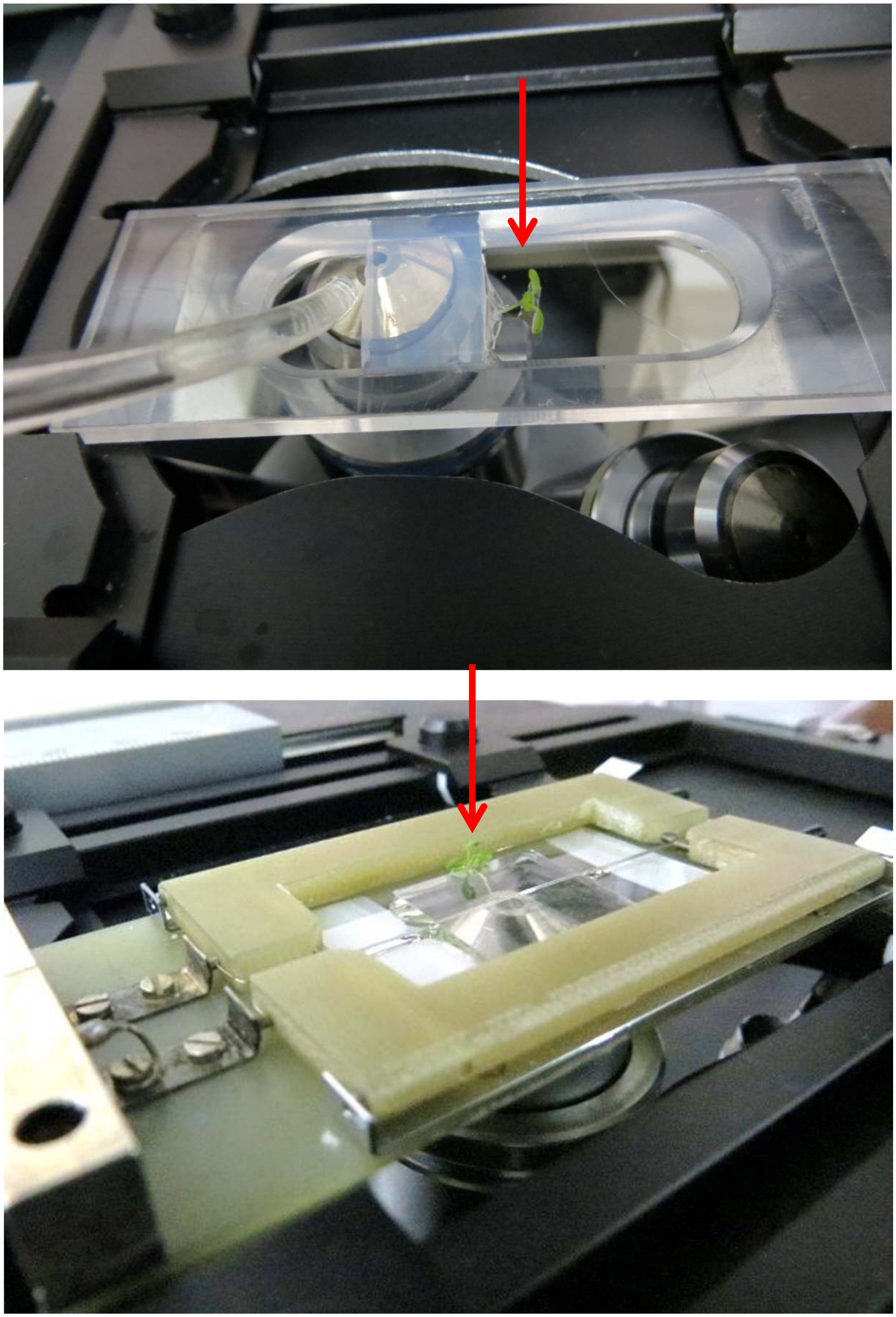

Figure S7, Matzke et al.

Figure S7: Photographs showing mounted seedlings for treatments with eATP, light and ITMV

(A) Seedling mounted in an open-top bath chamber under an agar block together with a tube for applying ATP solution positioned at the edge of the agar block. Seedlings mounted in this way were also illuminated by different wavelengths of light.

(B) Seedling mounted between platinum electrodes of the Bügelkammer covered with a cover slip for exposure to ITMV (induced transmembrane voltage).
